# Mutations in the Catalytic Loop HRD Motif Alter the Activity and Function of Drosophila Src64

**DOI:** 10.1371/journal.pone.0028100

**Published:** 2011-11-23

**Authors:** Taylor C. Strong, Gurvinder Kaur, Jeffrey H. Thomas

**Affiliations:** Department of Cell Biology and Biochemistry, Texas Tech University Health Sciences Center, Lubbock, Texas, United States of America; University of Dayton, United States of America

## Abstract

The catalytic loop HRD motif is found in most protein kinases and these amino acids are predicted to perform functions in catalysis, transition to, and stabilization of the active conformation of the kinase domain. We have identified mutations in a Drosophila *src* gene, *src64*, that alter the three HRD amino acids. We have analyzed the mutants for both biochemical activity and biological function during development. Mutation of the aspartate to asparagine eliminates biological function in cytoskeletal processes and severely reduces fertility, supporting the amino acid's critical role in enzymatic activity. The arginine to cysteine mutation has little to no effect on kinase activity or cytoskeletal reorganization, suggesting that the HRD arginine may not be critical for coordinating phosphotyrosine in the active conformation. The histidine to leucine mutant retains some kinase activity and biological function, suggesting that this amino acid may have a biochemical function in the active kinase that is independent of its side chain hydrogen bonding interactions in the active site. We also describe the phenotypic effects of other mutations in the SH2 and tyrosine kinase domains of *src64*, and we compare them to the phenotypic effects of the *src64* null allele.

## Introduction

Src is a member of the Src Family Kinases (SFKs), a group of nonreceptor tyrosine kinases that share a common structure consisting of an N-terminal myristoylation site, an SH3 domain, an SH2 domain, a tyrosine kinase domain and a C-terminal negative regulatory domain. Myristoylation of the SFK protein tethers it to the inner face of the plasma membrane, whereas the SH3 and SH2 domains mediate interactions with proline-rich recognition sequences and phosphotyrosine-containing sequences, respectively [1], [2]. Intramolecular interactions between the SH2 and SH3 domains and their recognition sites in the SFK hold the protein in a closed, inactive conformation. Release of these intramolecular interactions by dephosphorylation of the C-terminus or by SH2 binding of another phosphotyrosine protein leads to the adoption of an open, partially active conformation. Phosphorylation of the activation loop leads to the adoption of the fully active conformation. The SFK active site is located in a cleft between the N-terminal lobe and the C-terminal lobe of the kinase domain, where substrate, ATP and Mg^++^ cations bind. Two critical elements of the active site are the DFG motif-containing activation loop and the HRD motif-containing catalytic loop [3]. The HRD amino acids are thought to be involved in the reaction mechanism or the formation and stabilization of the active site [4]–[6].

SFKs have been shown to be involved in regulating the actin-based microfilament cytoskeleton [1], [7]. The Drosophila genome contains two genes that encode SFKs: *src42* and *src64* [8]. Both act in the remodeling of the microfilament cytoskeleton during dorsal closure [9], [10]. However, in ring canal growth in the egg chamber, *src64* seems to function independently of *src42* [9]. Ring canals are intercellular bridges linking the nurse cells to the developing oocyte, formed from the actin-rich arrested cleavage furrow that remains after incomplete cell division [11]. Src64 is required for ring canal growth [12]–[17].

*src64* is also required for microfilament contraction during the formation of the cellular blastoderm [18]. During early Drosophila embryogenesis, synchronized nuclear division proceeds without concomitant cell division. After nuclear division stops, a single layer of cells is formed by the simultaneous and uniform invagination of plasma membrane between the peripheral nuclei [19]. The leading edge of membrane invagination, the cellularization front, consists of stable infoldings of membrane called furrow canals surrounded by microfilaments [20]–[25]. Contractile tension in the microfilament network maintains uniform invagination of furrow canals during early cellularization, and constriction of microfilament rings partly closes cell bases during late cellularization [18], [26]. However, contraction of the microfilament network is not required for membrane invagination [18], [26]. *src64* mutant defects in both microfilament ring contraction and ring canal expansion are easily quantified, providing sensitive and effective means of assaying the biological function of *src64* [12], [18].

To understand the role of *src64* in regulating microfilament ring contraction during cellularization, we identified point mutations in the *src64* coding region. Of particular interest were mutations in each of the three highly conserved amino acids that constitute the HRD motif of the kinase domain catalytic loop. We analyzed the phenotypes caused by the mutation in the catalytic aspartate and the *src64* null allele and found that Src64 kinase activity is required for microfilament ring contraction. We also found that mutations in the histidine and arginine residues produced weaker cytoskeletal defects and lower reductions of kinase activity than expected. We discuss the implications of these results on the roles of the HRD amino acids in kinase domain activity and activation.

## Materials and Methods

### Genetics

Flies were cultured on a standard medium at 22.5°. Embryos were collected from yeasted apple juice/agar plates at 22.5° and counted during days three to ten. After forty hours, hatched and unhatched eggs were counted. OreR was used as the wild-type strain. *src64^Δ17^* and *src64^KO^* have been described [12], [14], [18]. Other alleles are described in Flybase [8]. *src64* point mutations were identified by mismatch between the wild-type sequence between primers in exon five (atcgacgacaccgagtccgattg) and exon eight (cgacgtgaccgagaaggactcgaagt) and the Zuker EMS-mutagenized lines [27] by the Seattle Targeting Induced Localized Lesions in Genomes (TILLING) Project [28]. The mutations consisted of one mutation in an intron, eight silent mutations, ten unique missense mutations, a duplicate of one of the missense mutations and two additional missense mutations that were no longer available. Potential background mutations were reduced by generating *th st cu sr e^s^ ca* recombinants. *src64^Δ17^*, *src64^KO^* and *src64^+^* were *th st cu sr e^s^ ca*. *src64^S440F^* recombinants were *st cu sr e^s^ ca*, and *src64^D372N^* and *src64^H402L^* recombinants were *h th st cu sr e^s^ ca*. Alleles were confirmed by PCR, polymorphism or direct sequencing.

### Immunohistochemistry and image analysis

Embryos were methanol heat-fixed [29] and stained with rabbit anti-myosin II heavy chain antibody (1∶1000) [30]. Ovaries were dissected and fixed in 4% paraformaldehyde [31] and stained with either anti-HTS antibody (1∶1) [11] (Developmental Studies Hybridoma Bank) or Alexafluor 488-conjugated phalloidin (Invitrogen) and Hoechst. Specimens were imaged using an Axioimager.A1 fluorescence microscope (Zeiss) or a Fluoview 300 confocal microscope (Olympus). Images were analyzed using Axiovision 4.4 (Zeiss) and ImageJ (W. Rasband, NIH; http://rsb.info.nih.gov/ij/). Outer diameters of all ring canals were measured in stage 10A egg chambers [12]. 4–14 egg chambers were compared using Kruskal-Wallis/Dunn's test. For amorph tests, the Wilcoxon-Mann-Whitney test was used, and 5–6 egg chambers for heterozygotes, and 11–19 egg chambers for homozygotes and *trans*-heterozygotes were analyzed. Cellularization microfilament rings from the embryo midsection were measured and circularity (4πA/p^2^ where A = area, and p = perimeter) calculated [18]. 10 rings from each of 3–6 embryos were compared using one-way ANOVA/Tukey-Kramer test. For amorph tests, the Wilcoxon-Mann-Whitney test was used on 25 rings from each of 5–10 embryos.

### Kinase assays

Homozygous *src64* mutant embryos from homozygous *src64* mutant parents were collected in a mesh basket and dechorionated in 50% bleach for two minutes [29] and washed in phosphate-buffered saline pH 6.5/0.1% Tween 20. Cellularizing embryos were selected by hand under the stereoscopic microscope, disrupted using a melted micropipette tip as a pestle in hypotonic lysis buffer [32] with 50% glycerol, and centrifuged at 16,000x g for 30 min at 4°. Supernatants were stored at −20°. Kinase assays were conducted on 0.58 µg to 1.80 µg of total protein using the SignaTECT protein tyrosine kinase assay system (Promega). Reactions proceeded in the presence of γ-^32^P-labeled ATP and sodium vanadate for 15 minutes at 30°. Phosphotransfer to the manufacturer's peptide substrate 2 was measured by liquid scintillation counting, and did not differ by cellularization stage. Src64-dependent tyrosine kinase activity was determined by subtracting measured total tyrosine phosphotransfer in *src64^KO^* mutant embryo extracts (7.97 pmol/min/mg) from measured total tyrosine phosphotransfer in the other samples. Five independent extracts were assayed from each genotype. *src64^KO^* and *src64^D404N^* extracts were generated by pooling supernatants from multiple embryo collections because of the extremely low numbers of embryos produced by these genotypes. Kinase assays were conducted in triplicate. Kinase activity was compared to wild type using ANOVA/Dunnett test.

## Results

### Identification of missense mutations in *src64*

To identify mutations that alter the coding sequence of *src64* in a phenotypically unbiased manner, mutants were screened using the Targeting Induced Localized Lesions in Genomes (TILLING) procedure [27], [28]. Ten missense mutations were identified: five in the SH2 domain and five in the tyrosine kinase domain (Table 1).

**Table 1:** *src64* mutations and fertility of *src64^KO^ trans*-heterozygous females.

Allele	Mutation^a^	Substitution^b^	Domain	Eggs laid/female	Eggs hatched (%)
*+*	-	-	-	498 (n = 4)	84% (n = 1992)
*src64^Δ17^*	5′ deletion [12]	-	-	120 (n = 9)	43% (n = 1017)
*src64^KO^*	Knockout [14]	-	-	13 (n = 17)	15% (n = 208)
*src64^P190L^*	CCT→CTT	P190L	SH2	216 (n = 9)	86% (n = 1853)
*src64^D204V^*	GAT→GTT	D204V	SH2	379 (n = 5)	74% (n = 1812)
*src64^G208E^*	GGA→GAA	G208E	SH2	390 (n = 5)	84% (n = 1905)
*src64^R217C^*	CGT→TGT	R217C	SH2	411 (n = 8)	87% (n = 2794)
*src64^C259Y^*	TGC→TAC	C259Y	SH2	322 (n = 8)	67% (n = 2124)
*src64^D372N^*	GAT→AAT	D372N	kinase	428 (n = 5)	87% (n = 2138)
*src64^H402L^*	CAT→CTT	H402L	kinase	323 (n = 6)	73% (n = 1796)
*src64^R403C^*	CGC→TGC	R403C	kinase	332 (n = 5)	88% (n = 1659)
*src64^D404N^*	GAT→AAT	D404N	kinase	93 (n = 10)	28% (n = 850)
*src64^S440F^*	TCC→TTC	S440F	kinase	412 (n = 9)	96% (n = 2042)

Mutation is shown for each allele. + indicates the wild-type allele. For point mutations, the base pair change, the amino acid substitution and the protein domain containing the substitution are shown. Female fertility was assayed in females *trans*-heterozygous for the mutation and the *src64^KO^* allele. Number of eggs laid by females over days 3–10 after introduction of males, is shown.

a Altered nucleotide is underlined.

b Wild-type amino acid is indicated, followed by position and mutant amino acid.

### Fertility and Embryonic Viability Defects

Most of the *src64* mutants can be maintained as homozygous lines. Neither the null allele *src64^KO^* [14] nor the *src64^D404N^* allele is viable as a homozygous line; however, heterozygous parents produce homozygous progeny that produce homozygous embryos that live past gastrulation. The nuclear defect in *src64^Δ17^* mutant embryos [18] was not observed in other *src64* mutants, suggesting that it is not caused by *src64* mutation.

Src64 protein is not expressed in the male reproductive system, and *src64^KO^* mutants have no defects in spermatogenesis or male fertility (A. O'Reilly, personal communication). However, *src64^Δ17^* and *src64^KO^* mutant females show fertility defects: reduced number of eggs laid and reduced proportion of eggs that hatch [12], [14]. We characterized female fertility defects by crossing wild-type males to females *trans*-heterozygous for *src64* mutations and *src64^KO^* to minimize potential fertility defects caused by background mutations, and scoring egg yield and hatch rate during the most robust period of egg production. Most, if not all, of the *src64* mutations caused lower egg yields (Table 1). The *src64^Δ17^*, *src64^KO^* and *src64^D404N^* mutations produced substantially lower egg yields than the wild-type allele (Table 1). Overall relative fecundity of mutant alleles is lower than indicated because egg laying drops off sooner in most mutants than in wild type. Fertility defects in *src64* mutant females are not caused by defects in the germline stem cells. Activated Src64 protein is not observed in either the germline stem cells or the somatic cells of the stem cell niche [14].

Maternal viability defects were milder than egg production defects. None were observed for *src64^P190L^*, *src64^G208E^*, *src64^R217C^*, *src64^D372N^*, *src64^R403C^* and *src64^S440F^*. The higher hatch rate of eggs laid by *src64^S440F^/src64^KO^ trans*-heterozygous females was likely caused by heterozygosity for *th*. *src64^KO^* and *src64^D404N^* mutations caused severely reduced hatch rates (Table 1). None of the females *trans*-heterozygous for *src64^KO^* showed an egg laying or hatching defect as severe as that of *src64^KO^* homozygous females, suggesting that these mutations are reduction-of-function mutations.

### Ring canal growth defects

**Figure 1:**
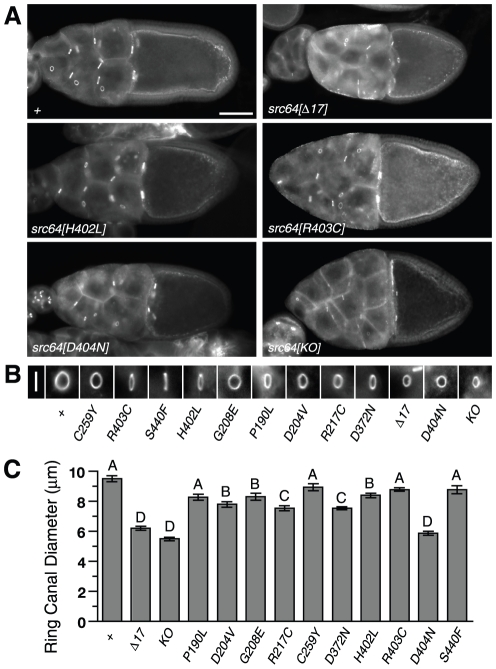
Ring canal growth defects in *src64* mutant egg chambers. Ring canals are stained with antibody to HTS. (A) Egg chambers. Ring canal diameters are reduced in the *src64^Δ17^*, *src64^H402L^*, *src64^D404N^* and *src64^KO^* mutants. Scale bar, 50 µm. (B) Typical ring canals ordered mean by diameter and oriented so diameter is presented along the vertical axis. Scale bar, 10 µm. (C) Ring canal diameters. A, does not differ from *+*; B, differs from *+* (P<0.05), but not other A alleles, except *src64^D204V^* differs from *src64^R403C^* (P<0.05); C, differs from *+* (P<0.001) and from A alleles (P<0.05) except *src64^P190L^*, but not from group B, except *src64^D372N^* differs from *src64^H402L^* (P<0.01); D, does not differ from *src64^KO^*, but differs from all other alleles (P<0.001). Error bars are SEM.

*src64* is required for the growth of the cytoskeletal ovarian ring canals in the Drosophila egg chamber [12]–[14]. We observed ring canal size defects in the egg chambers of most of the homozygous *src64* mutants; only *src64^P190L^*, *src64^C259Y^*, *src64^R403C^* and *src64^S440F^* were indistinguishable from wild type (Fig. 1). Ring canal sizes varied among the other *src64* mutants. Ring canal diameters of *src64^D204V^*, *src64^G208E^*, *src64^R217C^*, *src64^D372^* and *src64^H420L^* mutant egg chambers were significantly smaller than those of wild-type egg chambers (Fig. 1). *src64^Δ17^*, *src64^D404N^* and *src64^KO^* mutants had smaller ring canals than any of the mutants described above. The ring canal diameters of these mutants were indistinguishable (Fig. 1).

**Figure 2:**
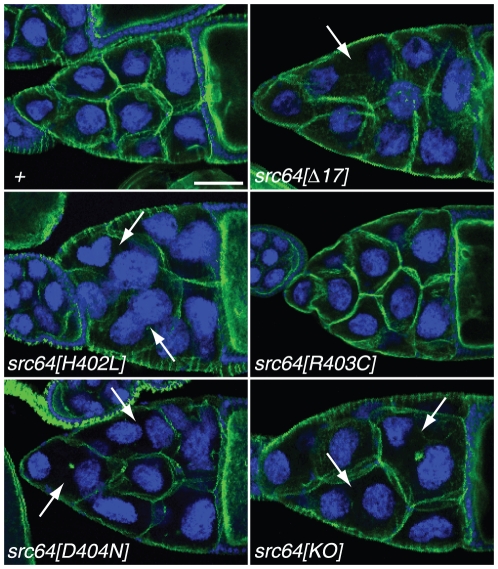
Nurse cell fusion defects in *src64* mutant egg chambers. Egg chambers are stained with phalloidin (green) and Hoechst (blue). Partial confocal projections are shown. Nurse cell fusion (arrows) occurs in *src64^Δ17^*, *src64^H402L^*, *src64^D404N^* and *src64^KO^* mutants. Nurse cell fusion does not occur in *src64^R403C^* mutants. Scale bar, 50 µm.

*src64* is also required for ring canal attachment to the nurse cell plasma membrane [12]. *src64^Δ17^* and *src64^KO^* mutants often have ring canal attachment defects and nurse cell fusion defects [12], [14]. *src64^D404N^* mutants have severe nurse cell fusion defects similar to those of *src64^KO^* mutants (Fig. 2). *src64^H402L^* mutants have nurse cell fusion defects that occur less frequently than those in *src64^Δ17^* mutants (Fig. 2). No nurse cell fusions were observed in *src64^R403C^* mutant egg chambers (Fig. 2). Detached and prematurely degenerating ring canals are sometimes observed in *src64* mutants [12]. We also occasionally observed degenerating ring canals in the egg chambers of strong *src64* mutants.

### Cellularization microfilament ring contraction defects

**Figure 3:**
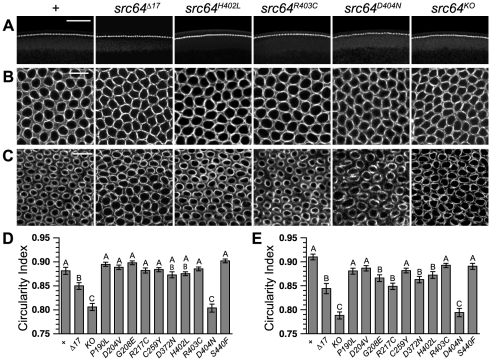
Cellularization microfilament defects in *src64* mutant embryos. Embryos are stained with antibody to myosin II heavy chain. (A) Cross-sections showing the early cellularization front. Furrow canal depths are less uniform, producing a wavy and irregular cellularization front in *src64^Δ17^*, *src64^H402L^*, *src64^D404N^* and *src64^KO^* mutant embryos. Scale bar, 50 µm. (B) Grazing section projections of early cellularization stage embryos showing the microfilament network. Microfilament rings of *src64^Δ17^*, *src64^H402L^*, *src64^D404N^* and *src64^KO^* mutant embryos are irregular. Scale bar, 10 µm. (C) Grazing section projections of late cellularization stage embryos showing microfilament rings. Microfilament rings of *src64^Δ17^*, *src64^H402L^*, *src64^D404N^* and *src64^KO^* mutant embryos are irregular and less constricted than the microfilament rings of wild-type embryos and *src64^R403C^* mutant embryos. Scale bar, 10 µm. (D) Microfilament ring circularity during early cellularization. A, does not differ from *+*; AB, differs from *+* and *src64^Δ17^*; B, does not differ from *src64^Δ17^* allele but differs from *+* (P<0.01); C, does not differ from *src64^KO^* but differs from *+* (P<0.001) and *src64^Δ17^* (P<0.001). Error bars are SEM. (E) Microfilament ring circularity during late cellularization. A, does not differ from *+*; B, does not differ from *src64^Δ17^* but differs from *+* (P<0.05); C, does not differ from *src64^KO^* but differs from *+* (P<0.001) and *src64^Δ17^* (P<0.001). Error bars are SEM.

*src64* is required for microfilament contraction during cellularization [18]. During early cellularization, microfilament rings are linked together, creating tension in the network through microfilament contraction [18]. We analyzed *src64* mutant embryos that are both maternally and zygotically mutant: homozygous for the *src64* mutation and derived from mothers homozygous for the *src64* mutation. Furrow canals do not invaginate uniformly during early cellularization in *src64^Δ17^* mutant embryos; variations in the depth of the furrow canals lead to the appearance of a wavy cellularization front (Fig. 3A) [18]. *src64^H402L^, src64^D404N^* and *src64^KO^* mutant embryos show this phenotype (Fig. 3A), as do *src64^R217C^* and *src64^D372N^* mutant embryos. The cellularization front defects of *src64^R217C^, src64^D372N^* and *src64^H402L^* mutant embryos were weak and incompletely penetrant, similar to *src64^Δ17^* mutant embryos (Fig. 3A). The defect was severe in both *src64^D404N^* and *src64^KO^* mutant embryos (Fig. 3A). None of the mutants showed temperature-sensitive defects.

To assay microfilament ring contraction defects, we determined the circularity index, a normalized ratio of area to perimeter. Deviation from circularity suggests a reduction of contractile tension in the microfilament ring [18]. Most of the *src64* mutations do not cause microfilament ring defects during early cellularization. Although some *src64^R217C^*, *src64^D372N^* and *src64^H402L^* mutant embryos have early cellularization front defects, they do not have microfilament ring circularity defects (Fig. 3B, D). The microfilament ring circularity defects of *src64^D404N^* and *src64^KO^* mutant embryos are indistinguishable, and significantly more severe than that of *src64^Δ17^* mutant embryos (Fig. 3B, D).

During late cellularization, microfilament rings constrict [18], [20]. Microfilament rings in *src64^G208E^*, *src64^R217C^*, *src64^D372N^* and *src64^H402L^* mutant embryos show little constriction, similar to that observed in *src64^Δ17^* mutant embryos, and differ in circularity from wild-type embryos (Fig. 3C, E). The microfilament rings of *src64^KO^* and *src64^D404N^* mutant embryos do not noticeably constrict and have more severe circularity defects than *src64^Δ17^* mutant embryos (Fig. 3C, E).

### Catalytic loop HRD mutations reduce or eliminate Src64 kinase activity

Three mutations, *src64^H402L^*, *src64^R403C^* and *src64^D404N^*, alter the three conserved amino acids of the catalytic loop HRD motif. To analyze the biochemical effects of these mutations, we conducted kinase assays on extracts from cellularizing embryos. Extracts from wild-type embryos and *src64^KO^* mutant embryos were used as controls for full tyrosine kinase activity and Src64-independent activity, respectively. The majority of tyrosine phosphotransfer to the peptide substrate is Src64-dependent. Much of the Src64-independent phosphotransfer is likely due to the activity of Src42, an SFK present during cellularization but primarily localized to a different subcellular domain [9], [18]. Most Src64-dependent tyrosine phosphotransfer is likely to be directly catalyzed by Src64, but may include phosphotransfer from any other tyrosine kinases activated by Src64.

**Figure 4:**
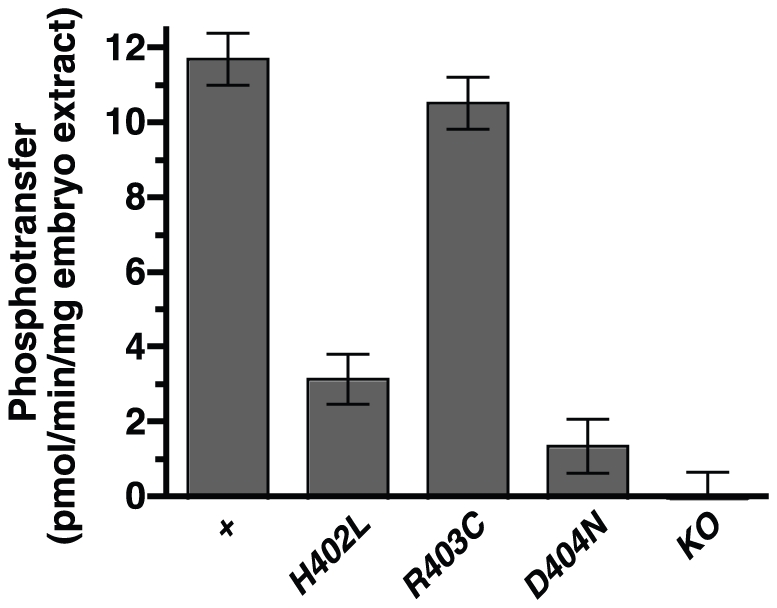
Tyrosine kinase activities of *src64* HRD mutants. Phosphotransfer was measured in cellularization-stage embryo extracts. Src64-dependent phosphotransfer to a peptide substrate is shown. *src64^H402L^*, *src64^D404N^* and *src64^KO^* differ from wild type (p<0.01). Wild type, *src64^H402L^* and *src64^R403C^* differ from *src64^KO^* (p<0.01). Error bars are SEM.

We compared the Src64-dependent tyrosine kinase activities of the HRD mutants to those of the controls. *src64^H402L^*, *src64^R403C^* and *src64^D404N^* mutant embryo extracts had 27%, 90% and 12% of wild-type activity, respectively (Fig. 4). Tyrosine kinase activity of the mutant Src64^R403C^ protein is indistinguishable from that of the wild-type Src64 protein (Fig. 4). This is consistent with the wild-type cellularization front and microfilament contraction phenotypes observed in *src64^R403C^* mutant embryos (Fig. 3). Tyrosine kinase activity of the mutant Src64^D404N^ protein is indistinguishable from Src64-independent activity (Fig. 4). This is consistent with the observation that the *src64^D404N^* cellularization phenotypes are indistinguishable from those of *src64^KO^* (Fig. 3). The Src64^H402L^ mutant protein shows intermediate tyrosine kinase activity (Fig. 4), consistent with the weak to moderate phenotypes observed in *src64^H402L^* mutant embryos during cellularization (Fig. 3).

### *src64^D404N^* is a loss-of-function mutation for ring canal growth and microfilament contraction

**Figure 5:**
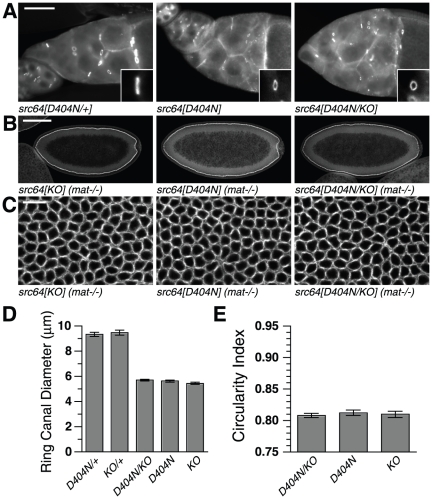
Cytoskeletal defects of *src64^D404N^/src64^KO^ trans*-heterozygotes. (A) *src64^D404N^/+*, *src64^D404N^* and *src64^D404N^/src64^KO^* egg chambers stained with antibody to HTS. Ring canal diameters are not reduced in *src64^D404N^/+* egg chambers but are reduced in *src64^D404N^/src64^KO^* egg chambers. Inset: ring canal of approximately mean diameter, reoriented so that diameter is projected along the vertical axis. Scale bar, 50 µm. (B) Early cellularization stage embryos laid by *src64^KO^*, *src64^D404N^* and *src64^D404N^/src64^KO^* mothers. Embryos show non-uniform cellularization fronts. Scale bar, 100 µm. (C) Grazing section projections of early cellularization stage embryos laid by *src64^KO^*, *src64^D404N^* and *src64^D404N^/src64^KO^* mothers showing microfilament networks. Microfilament ring defects of maternally *trans*-heterozygous *src64^D404N^/src64^KO^* embryos are similar to those of *src64^KO^* and *src64^D404N^* embryos. Embryos are stained with antibody to myosin II heavy chain. Scale bar, 10 µm. (D) Ring canal diameters. *src64^D404N^* and *src64^D404N^/src64^KO^* do not differ (p = 0.35). Error bars are SEM. (E) Microfilament ring circularity during early cellularization. *src64^D404N^* and *src64^D404N^/src64^KO^* do not differ (p = 0.52). Error bars are SEM.

Since *src64^D404N^* mutants show strong defects in the egg chamber and in cellularizing embryos, we tested whether *src64^D404N^* is a loss-of-function allele for these cytoskeletal processes by conducting *trans*-heterozygote tests using the *src64^KO^* allele to eliminate *src64* function. The diameters of ring canals from s*rc64^D404N^/+* females and *src64^KO^/+* females were indistinguishable (Fig. 5A, D) and were similar to those of wild-type homozygotes (Fig. 1), indicating that *src64^D404N^* is a recessive allele. No significant differences were observed between ring canal diameters from *src64^D404N^/src64^KO^ trans*-heterozygous, *src64^D404N^* and *src64^KO^* egg chambers (Fig. 5A, D). These results suggest that *src64^D404N^* acts as a loss-of-function allele for ovarian ring canal growth.

We also tested the *src64^D404N^* allele during early cellularization. *src64* is maternally required for microfilament ring contraction during cellularization [33]. Maternally mutant embryos were constructed by crossing wild-type males to females of genotypes *src64^D404N^/src64^KO^*, *src64^D404N^* and *src64^KO^*. The cellularization front defect of the *src64^D404N^/src64^KO^ trans*-heterozygous embryos was similar to those of the homozygous embryos (Fig. 5B). The circularity defects were indistinguishable (Fig. 5C, E), suggesting that *src64^D404N^* acts as a loss-of-function allele for microfilament ring contraction during cellularization.

## Discussion

### *src64* ^D404N^ mutation

The catalytic loop aspartate at position 404 in Src64 (chicken c-Src D386) has a critical role in catalysis [3], [5]. It has been proposed to act as a catalytic base, deprotonating the tyrosine hydroxyl to catalyze a nucleophilic attack on the γ-phosphate group of ATP as part of the phosphoryl transfer reaction. However, many studies suggest that the neutral hydroxyl group acts as the nucleophile. Aspartate hydrogen bonds, directly or indirectly, to the hydroxyl group to position it for effective nucleophilic attack and acts as a proton acceptor late in the reaction [4], [5], [34]. In addition, the HRD aspartate may help stabilize the inactive state through an interaction with the unphosphorylated tyrosine in the activation loop [35].

Yeast carrying an aspartate to alanine substitution in cAMP-dependent protein kinase (PKA) were nearly inviable and had only 0.4% of the kinase activity of wild type [36]. Similarly, phosphorylase kinase protein (Phk) with this mutation showed little activity. Substitution with asparagine, the neutral amide derivative of aspartate, eliminates charge without altering hydrogen-bonding interactions that do not involve the carboxyl group. This mutation strongly reduced Phk kinase activity, but not as strongly as alanine. It caused a relatively small reduction in ATPase activity, suggesting that aspartate is critical for phosphoryl transfer rather than ATP hydrolysis [37]. The asparagine mutation in the tyrosine kinase Csk also strongly reduces, but does not eliminate, kinase activity [38]. Mutation to glutamate alters structure and size but not charge; this mutation also greatly reduces Csk activity [39].

Here we describe the effect of the HRD aspartate to asparagine mutation on several well-characterized and quantifiable biological processes in a living organism. Kinase activity of the *src64^D404N^* mutant is indistinguishable from that of *src64^KO^*. *src64^D404N^* behaves like a null allele in both cytoskeletal processes examined. Therefore, the *src64^D404N^* mutation either eliminates kinase activity or reduces it below the threshold required for function. However, for fertility and embryonic viability, *src64^D404N^* acts as a reduction-of-function allele. Low levels of Src64 kinase activity may be sufficient for some biological function. Together, these results are consistent with *in vitro* studies of purified PKA, Phk and Csk proteins showing that mutation to asparagine severely reduced, but did not eliminate, kinase activity. Alternately, *src64^D404N^* may behave as a reduction-of-function mutation in fertility because Src64 has a kinase-independent activity; however, kinase-dead SFKs can titrate negative regulators and increase the activity of other SFKs, arguing against this [40], [41]. Although kinase-inactive alleles of *src* have been reported to act as dominant-negative alleles [42]–[44], we did not observe dominant negative activity of *src64^D404N^*. When over-expressed in the developing eye, kinase-dead SFKs also do not act in a dominant negative manner [40].

### *src64* ^R403C^ mutation

RD kinases such as SFKs have arginine in the catalytic loop HRD motif and are only fully activated by phosphorylation of the activation loop [45]. Transition to the fully active conformation is salt-dependent and the HRD arginine has different charge-based interactions in the active and inactive conformations, suggesting that activation involves an electrostatic switch [46]–[50]. In the inactive conformation, glutamate E327 (c-Src E310) is salt-bridged to either the HRD arginine R403 (c-Src R385) or the activation loop arginine R427 (c-Src R409) [48], [49]. In the active conformation, the phosphorylated tyrosine interacts with both R403 and R427, and glutamate is salt-bridged to lysine K312 (c-Src K295), a critical linkage in the fully active conformation [48], [51]–[53]. The arginine switch may be part of a sequential series of electrostatic switches that drive conformation change [35], [50]. These arginines are likely partially redundant for phosphotyrosine coordination: the activation loop arginine plays the major role in stabilizing both active and inactive configurations in SFKs, whereas the HRD arginine plays the major role in serine/threonine kinases [35], [53], [54]. The HRD arginine may also stabilize the DFG aspartate in the active state [55].

Mutation of the HRD arginine to alanine in yeast PKA reduces kinase activity to 10.5% of wild-type activity, but viability is unaffected [36]. In PhK, this mutation also reduces kinase activity [37]. Kinase activity of the chicken c-Src mutant protein is 10% for an exogenous substrate but 50% for autophosphorylation [56].

Mutation of the HRD arginine to cysteine has remarkably little effect on Src64. Kinase activity was indistinguishable from wild type, and there was no effect on microfilament contraction, ring canal growth or hatching and only weak defects in egg production. Our data do not support a major role for the HRD arginine in stabilization of the active site or in an electrostatic switch for activation, and are consistent with the activation loop arginine being the critical arginine residue in tyrosine kinases. However, the Src64 cysteine mutant has more kinase activity than the c-Src alanine mutant, suggesting that cysteine may not abrogate biochemical activity. The alanine side chain is short and aliphatic, so it likely eliminates biochemical activity, whereas the polar cysteine side chain may retain sufficient biochemical activity for function. Alternately, *src64^R403C^* may destabilize both the active and inactive conformations by failing to interact with both phospho-Y434 and E327, thus favoring the partially active conformation that would produce a constant but low level of activity.

### *src64* ^H402L^ mutation

The HRD histidine is, along with the HRD aspartate, one of ten critical residues conserved amongst eukaryotic protein kinases and eukaryotic protein kinase-like kinases in prokaryotes [57]. The histidine peptide backbone hydrogen bonds to the C-terminal end of the activation loop DFG motif and to the F-helix aspartate in the kinase domain C-terminal lobe. The side chain has a hydrophobic packing interaction with the DFG phenylalanine, and hydrogen bonds to the N-terminal end of the DFG motif and to the polypeptide backbone of the HRD aspartate. Thus, the HRD histidine provides two critical links in the active kinase: between the catalytic and activation loops in the active site, and between the active site and the enzyme core [6]. The histidine side chain is a component of the regulatory spine (R-spine), a stack of amino acid side chains linked by hydrophobic interactions that spans the fully active kinase domain [58], [59]. The R-spine consists of four residues: the catalytic loop HRD histidine H402 (c-Src H384), the activation loop DFG phenylalanine F423 (c-Src F408), a C-helix hydrophobic residue M331 (c-Src M317), and a β-4 strand leucine L342 (c-Src L328) [58]–[61]. The hydrogen bond between the histidine polypeptide backbone and the F-helix aspartate anchors the R-spine to the rigid F-helix. Together with the catalytic spine (C-spine), another stack of hydrophobic residues anchored to the F-helix, it connects the two lobes of the kinase domain and stabilizes the active conformation [6], [58], [59], [61].

The HRD histidine has not been investigated by *in vitro* mutagenesis, but the other three R-spine residues have. Mutation of the p38 MAP kinase DFG phenylalanine to alanine, arginine, or glycine abrogated activity, whereas mutation to tyrosine reduced kinase activity to 1% [55]. The mutant tyrosine side chain retained its hydrophobic contact with histidine, suggesting that addition of a polar hydroxyl group is sufficient to partially destabilize the R-spine [55], [62]. Mutation of either the DFG phenylalanine or the C-helix methionine to glycine rendered the nonreceptor tyrosine kinase Abl inactive, whereas mutation of the β-4 strand leucine residue to glycine reduced kinase activity [60].

Here we describe the effect of an HRD histidine mutation on the activity and function of a kinase in a living organism. The *src64^H402L^* mutation reduces kinase activity to 27% of wild-type activity and produces moderate to weak, but significant, defects in microfilament ring contraction and ring canal growth. Therefore, a quarter of the wild-type level of Src64 activity is not sufficient for normal cytoskeletal function in these processes. However, substantial cytoskeletal function remains at this lower Src64 activity level. We postulate that the *src64^H402L^* mutation alters interactions within the active site without altering the global conformation of the active kinase. The mutant leucine side chain is unlikely to hydrogen bond with the polypeptide backbones of the DFG aspartate and the HRD aspartate; loss of these active site interactions likely causes the reduction in kinase activity and the phenotypic defects. The hydrophobic interaction with the DFG phenylalanine should be retained, so a stable R-spine should still form. The hydrogen bond from the polypeptide backbone to the F-helix aspartate should be retained, so the catalytic loop and the R-spine should still be anchored to the F-helix, and thus linked to the C-lobe and the C-spine. Likewise, the hydrogen bond from the polypeptide backbone to the DFG motif is unlikely to be altered. Therefore, several interactions between histidine and the activation loop should be retained. The phenotype of this mutant suggests that the primary function of the HRD histidine may be to assemble and stabilize the R-spine and anchor it to the F-helix, and that its role in linking the catalytic loop to the activation loop may be less important.

In summary, our data suggest that the catalytic loop HRD amino acids have more complex roles in kinase activity and activation than previously thought. In addition, we have developed an experimental paradigm for a combined *in vivo* and *in vitro* investigation of the roles of specific amino acids in Src64. By making specific amino acid substitutions in a *src64* transgene expressed in *src64^KO^* flies, this approach can be used to further define the biochemical roles of the HRD histidine and arginine amino acids in SFKs.
